# Improved survival outcome with not-delayed radiotherapy and immediate PD-1/PD-L1 inhibitor for non-small-cell lung cancer patients with brain metastases

**DOI:** 10.1007/s11060-023-04459-4

**Published:** 2023-10-17

**Authors:** Yaner Yu, Haiyan Chen, Zhifeng Tian, Qun Zhang, Yongjie Shui, Li Shen, Qiongge Hu, Zhifei Huang, Shuangqiu Zhu, Hao Jiang, Qichun Wei

**Affiliations:** 1 Department of Radiation Oncology (Key Laboratory of Cancer Prevention and Intervention, China National Ministry of Education, Key Laboratory of Molecular Biology in Medical Sciences, Zhejiang Province, China), The Second Affiliated Hospital, Zhejiang University School of Medicine, Hangzhou, Zhejiang China; 2 Cancer Institute (Key Laboratory of Cancer Prevention and Intervention, China National Ministry of Education, Key Laboratory of Molecular Biology in Medical Sciences, Zhejiang Province, China), The Second Affiliated Hospital, Zhejiang University School of Medicine, Hangzhou, Zhejiang China; 3 Zhejiang Provincial Clinical Research Center for Cancer, Cancer Center of Zhejiang University, Hangzhou, China; 4 Department of Radiotherapy, Lishui Municipal Central Hospital, Lishui, China; 5 Anhui Campus of the Second Affiliated Hospital, Zhejiang University School of Medicine, Bengbu, 233000 China; 6 Department of Radiation Oncology, The First Affiliated Hospital of Bengbu Medical College, Bengbu, 233000 China

**Keywords:** Non-small-cell lung cancer, Brain metastasis, Radiotherapy, Immunotherapy, Sequencing

## Abstract

**Purpose:**

To investigate the impact of radiotherapy (RT) and immune checkpoint inhibitor (ICI) sequence on the survival outcome in NSCLC patients with brain metastasis, and decide the best time to initiate RT.

**Methods:**

Patients were managed with delayed RT (ICI delivered over 2 weeks prior to RT), concurrent RT (ICI delivered within 2 weeks prior to or after RT), or upfront RT (RT delivered over 2 weeks prior to ICI). Overall survival (OS), intracranial local progression-free survival (iLPFS), and intracranial distant progression-free survival (iDPFS) were assessed. A meta-analysis was performed to analyze the association between survival outcome and RT/ICI sequence.

**Results:**

A total of 73 NSCLC patients were identified with a median follow-up of 13.9 months. Patients who receive delayed RT demonstrated shorter iLPFS (P = 0.0029), iDPFS (P = 0.016), and OS (P < 0.001). A meta-analysis was conducted and a total of 4 studies, 254 patients were included. The HR was 0.44 for iDPFS (P = 0.03), 0.41 for OS (P < 0.01) when compared concurrent with delayed RT, 0.21 for iDPFS (P < 0.01), 0.32 for OS (P < 0.01) when compared upfront with delayed RT, consistent with our conclusion that delayed RT brought with worst iDPFS and OS. More importantly, the best overall response rate (BOR) decreased in cases with longer RT and ICI intervals. Patients who receive intervals of RT and ICI within 7 days achieve the best median BOR of − 53%.

**Conclusions:**

Delayed RT brought poor survival outcomes including iLPFS, iDPFS, and OS in NSCLC patients. The shorter interval of RT and ICI is associated with better BOR.

**Supplementary Information:**

The online version contains supplementary material available at 10.1007/s11060-023-04459-4.

## Introduction

Immune checkpoint inhibitor(ICI) has greatly improved the survival of patients with locally advanced non-small-cell lung cancer(NSCLC) [1]. The response rate to ICI is only between 10% and 25%, and the issue of primary or acquired resistance has become critical [2, 3]. An established method to overcome immunotherapy resistance in locally advanced and metastasized lung cancer is combined ICI with radiation, often known as iRT [4, 5]. But there are still a lot of unanswered problems, and one of the most hotly contested topics right now is the sequencing of iRT.

Sequential iRT has been adopted for most prospective studies (CTR20200299, Skyscraper-03, PACIFIC-3, GEMSTONE-301, et al.) since the PACIFIC study [4]. It is a method for administering ICI after chemoradiotherapy is finished. However, subgroup analysis revealed early initiation of ICIs (within 14 days following RT) predicts better overall survival(OS) and progression-free survival (PFS), and prompts the forward lead of immunotherapy [6]. To investigate the survival advantage of concurrent iRT, active prospective clinical trials are being conducted (KEYNOTE-799, Checkmate73L, KEYLYNK-012, Advan TIG-301). Which approach is superior is still an open question. In addition, the idea of “concurrent iRT” is under dispute, with some research utilizing a “2-week” window and others extending to a “1-month” frame.

Another conflict is to choose between delayed RT and upfront RT. Mechanistically, RT functions as an antigenic primer and has the potential to eradicate inhibitory T-cells in the tumor microenvironment [7, 8]. This is why the initiation of RT ahead is recommended. However, a greater partial response rate and OS advantage were found in the upfront ICI group in several trials [9, 10]. Recent studies also revealed increase in activated memory CD4+ and CD8+T cells was only detected after stereotactic ablative radiotherapy to parenchymal sites, it reminded us that immunomodulatory effect may be affected by radiation sites [11]. Due to above debates, the need to investigate the ideal iRT sequence has arisen.

BM patients represent a specific population with unique cell types, anatomical structures, metabolic constraints, and immune environments. Until recently, studies showed that ICI might cross the blood–brain barrier and increase the sensitivity of brain RT. A meta-analysis including BM from lung cancer patients, found ICI addition to intracranial RT is associated with improved OS (HR = 0.54, 95% CI 0.44–0.67; P < 0.001) [12]. To date, very few studies have described the ideal order of iRT to BMs in NSCLC. Indeed, there is a preference to withhold radiotherapy and initiate ICIs early in clinical practices, intending to obtain a better response rate and delaying radiotherapy as salvage treatment [13]. Concrete evidence for or against this practice is limited. Besides, the sequence of cranial RT and ICI may affect both efficacy and toxicity [10]. In order to determine the best time to start immunotherapy, we retrospectively evaluate the impact of RT and ICI timing in BM patients with NSCLC.

## Materials and methods

This multicenter retrospective cohort study included patients with NSCLC patients with BMs in the Second Affiliated Hospital of Zhejiang University and the First Affiliated Hospital of Bengbu Medical College from 2018 to 2022. The following were the inclusion requirements: (1) Patients receive ICI within 6 months of brain RT. (2) At least 1 month of brain magnetic resonance imaging (MRI) follow-up after brain RT. Patients with the following traits were disqualified: (1) People who have had preventative cranial radiation therapy. (2) Patients treated with systemic therapy during the period after RT and before initiation of ICI. (3) Any patients who received ICI on active or unreported clinical trials. In this study, the most commonly used ICIs were Pembrolizumab, Camrelizumab, Tislelizumab, and Sintilimab. A consensus was reached after two radiologists independently reviewed all radiological images.

We stratified patients into three groups according to the treatment sequence (Supplementary Fig. 1): In the concurrent group, ICI was given within 2 weeks before or after RT. In the upfront RT group, RT was completed at least 2 weeks before the start of the ICI. In the delayed RT group, RT was done at least 2 weeks after the last course of ICI. Not-delayed RT group refers to the combination of the concurrent group and upfront RT group. In this study, outcomes include intracranial local progression-free survival (iLPFS), intracranial distant progression-free survival (iDPFS), and Overall survival (OS). iLPFS extended from the date of RT initiation to local progression of BM inside PTV. iDPFS extended from the date of RT initiation to the earliest date of regional progression (defined as new BM, or progression of BM without radiotherapy). OS was computed starting from the date of RT until the last follow-up or until the death. We followed the RANO criteria for assessing progression and the response to treatment of brain metastasis lesions, with patients undergoing regular follow-up visits as proposed by clinical guidelines [14]. We also record the magnitude of the best objective response (BOR) of lesions measurable on MRI [13], relative to the pre-RT maximum diameter. We also calculated the size of tumor, which is defined as the sum of the largest diameter of measurable tumors prior to radiotherapy [15].

The methodology detailing evidence acquisition and data extraction for meta-analysis is detailed in Supplementary Text (1), Supplementary Fig. 2 is the PRISM Flow Diagram for the meta-analysis. The methodology detailing the statistical analysis is presented in Supplementary Text (2) This project was approved by the Independent Ethics Committee of the Second Affiliated Hospital of Zhejiang University and the First Affiliated Hospital of Bengbu Medical College.

## Results

### Characteristics of the included patients treated with ICIs and intracranial RT

A total of 73 consecutive NSCLC patients with BMs who received brain RT combined with PD-1/PD-L1 inhibitor were analyzed. Patients’ characteristics are summarized in Table 1. Forty-four patients (60.3%) received concurrent ICI and RT, seventeen patients (23.3%) were treated with delayed RT, and twelve patients (16.4%) received upfront RT. There was no difference between groups in terms of gender, age, smoking status, pathological type, ECOG performance status, BM number, DS-GPA, intracranial surgery, tumor size, neurological symptoms, or type of radiation at the time of brain metastases. The median interval between ICI and RT among patients who receive delayed RT was 2.06 months (95% CI = 1.40–2.71), and 3.20 months (95% CI = 2.04–4.54) among patients who receive upfront RT.

**Table 1:** Clinical baseline characteristics and treatment outcomes of enrolled patients

	Delayed RT (n = 17)	Concurrent RT (n = 44)	Upfront RT (n = 12)	p
Gender				
Male	13	29	9	0.575
Female	4	15	3	
Age (Median)				
< 61 years	9	21	9	0.268
≥ 61 years	8	23	3	
Smoking				
Non-smokers	6	18	4	0.770
Smokers	11	26	8	
Upfront intracranial surgery				
Yes	1	9	5	0.056
No	16	35	7	
Radiation type				
SRS	7	26	5	0.448
WBRT	3	6	4	
WBRT+SRS	7	12	3	
ECOG score				
0	2	10	2	0.102
1	7	26	5	
2	8	8	5	
Number of BMs				
> 2	9	14	4	0.430
≤ 2	8	30	8	
Neurological symptoms				
Yes	6	27	3	0.125
No	11	17	9	
Type of immunotherapy				
PD-1	17	43	12	0.711
PD-L1	0	1	0	
Median tumor size (mm, 95% CI)	23.2 (12.8–32.5)	21.9 (17.5–26.4)	26.0 (14.1–37.9)	0.740
ICI–RT interval (months, 95% CI)	2.06 (1.40–2.71)		3.30 (2.04–4.54)	
Response rate				
CR	4	6	2	0.717
PR	5	22	7	
SD	3	15	2	
PD	5	2	1	
Radiation necrosis				
Yes	2	6	2	0.934
No	15	36	10	
Median iLPFS (months, 95% CI)	11.18 (2.89–19.47)	23.8 (16.53–31.08)	17.31 (11.39–23.28)	**0.017**
Median iDPFS (months, 95% CI)	18.85 (11.56–26.15)	28.1 (21.84–34.37)	20.23 (15.20-25.27)	0.067
Median OS (months, 95% CI)	14.22 (7.43–21.01)	25.21 (18.97–31.45)	28.02 (18.3-37.74)	**0.001**

Bold values means variables with siginificant difference among three groups (P < 0.05）

### Delayed RT is a predictor of shortened intracranial local and distant PFS

The median follow-up from the initial RT of the study is 13.9 months (95% CI: 11.74–16.11 months). Six-month iLPFS and iDPFS rates for concurrent RT, delayed RT and upfront RT are 88.4%, 53.6%, 80.8% and 89.2%, 90.0%, 59.3%. Twelve-month iLPFS and iDPFS rates for concurrent RT, delayed RT and upfront RT are 73.1%, 26.8%, 60.6% and 89.2, 90.0%, 59.3%, respectively.

**Fig. 1:**
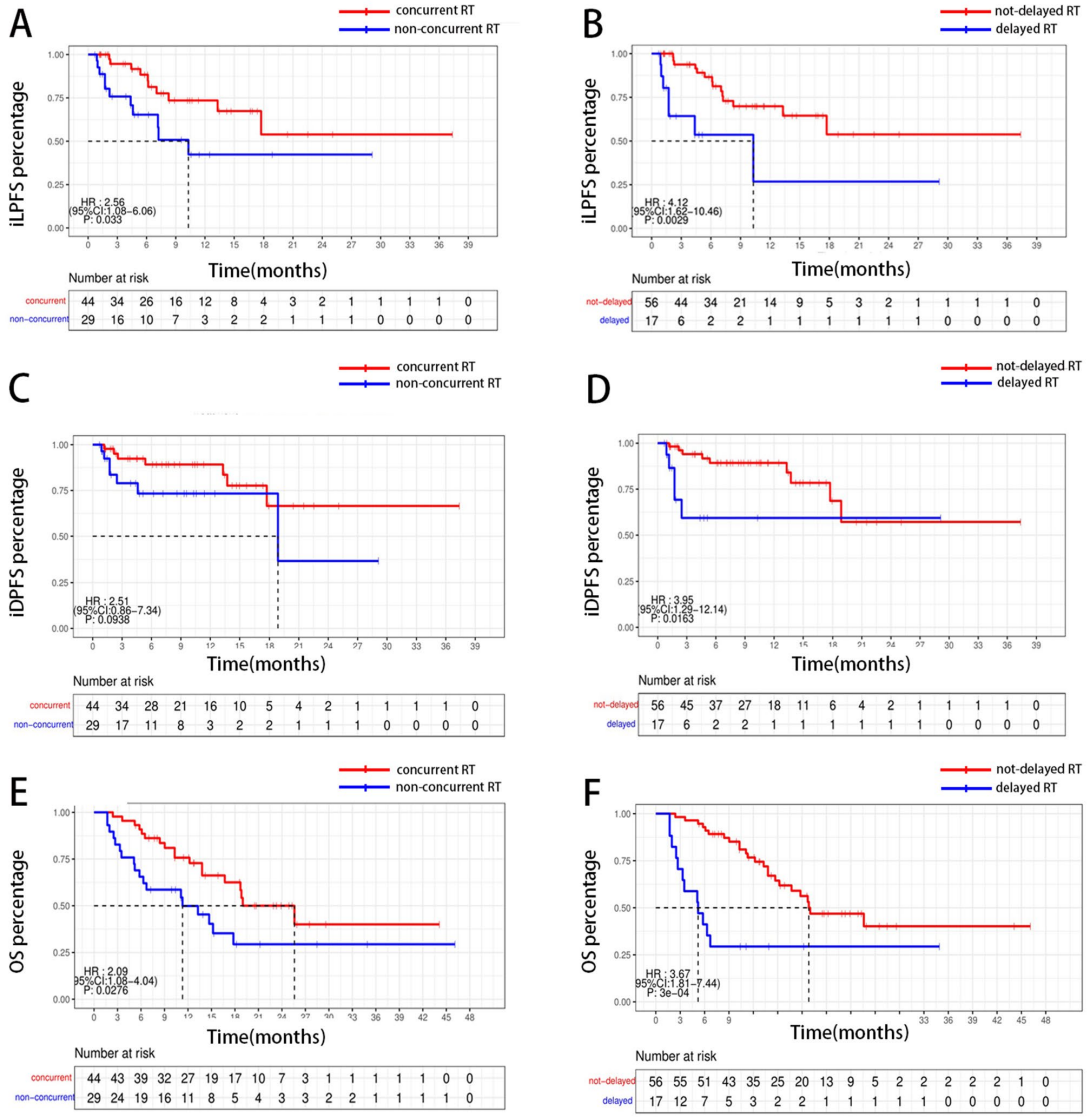
RT and ICI sequence is associated with survival outcome. Concurrent RT is a predictor of improved iLPFS(A), iDPFS(C), OS(E), patients administrated with delayed RT has worse iLPFS(B), iDPFS(D), OS(F)

As is shown in Fig. 1, concurrent delivery of RT and ICI was significantly associated with improved iLPFS compared to non-concurrent groups (patients who are administrated with delayed RT or upfront RT) (HR = 2.56, 95% CI = 1.08–6.06, P = 0.0033). When comparing delayed RT group vs. not-delayed RT groups, a significant difference was noted with median iLPFS of 11.51 mo vs. 24.37 mo, respectively (HR = 4.12, 95% CI = 1.62–10.46, P = 0.003) (Fig. 1**)**. Multivariate regression analysis (MVA) revealed the delayed RT was the only factor associated with a higher probability of intracranial progression **(**Table 2**)**.

**Table 2:** Cox regression analysis was used to determine predictive factors for iLPFS (A) and iDPFS (B)

Characteristics for Cox regression analysis	iLPFS				iDPFS			
	Univariate cox regression		Multivariate cox regression		Univariate cox regression		Multivariate cox regression	
	HR (95% CI)	p	HR (95% CI)	p	HR (95% CI)	p	HR (95% CI)	p
Gender (reference: female)	1.35 (0.52–3.49)	0.536			1.11 (0.36–3.41)	0.853		
Age (reference: ≤ 65) > 65 vs. ≤ 65 years	1.5 (0.6–3.7)	0.384			2.33 (0.73–7.41)	0.153		
Type of ICI treatment (reference: PD-L1) PD-1 vs. PD-L1	3349879.28 (0-lnf)	0.998			3350308.98 (0-lnf)	0.998		
Prior brain surgery (reference: no) Yes vs. no	1.29 (0.5–3.35)	0.602			0.84 (0.23–3.04)	0.786		
Number of BM (reference: ≤ 2 BM) > 2 vs. ≤ 2	2.02 (0.86–4.77)	0.109			**2.48 (0.85–7.2)**	**0.095**	1.8 (0.74–4.35)	0.192
Metastasis excluding brain (reference: no)	0.82 (0.28–2.37)	0.714			1.49 (0.37–5.99)	0.572		
ECOG score (reference: ECOG = 0) 2 vs. 0 1 vs. 0	**3.86 (1.17–12.72)** **3.36 (1.14–9.92)**	**0.026** **0.029**	1.44 (0.54–3.86) 1.24 (0.52–2.92)	0.464 0.625	**10.91 (1.33–89.2)** **16.78 (2.01**–**140.22)**	**0.026 0.009**	1.37 (0.57–3.3) 1.75 (0.63–4.87)	0.480 0.287
Neurological symptoms (reference: no) Yes vs. no	1.43 (0.57–3.56)	0.447			1.47 (0.49–4.44)	0.491		
Ds-GPA (reference:0–1) 1.5-2 vs. 0–1	0.95 (0.38–2.38)	0.920	0.7 (0.31–1.55)	0.374	**0.43 (0.14–1.37)**	**0.154**	1.04 (0.39–2.79)	0.941
**2.5–4 vs. 0–1**	**0.22 (0.05–1.05)**	**0.057**	**0.23 (0.06–0.86)**	**0.028**	**0.19 (0.04–0.98)**	**0.047**	0.41 (0.09–1.94)	0.261
Type of Radiation								
WBRT+SRS vs. SRS	0.95 (0.37–2.44)	0.920			0.91 (0.29–2.8)	0.865		
WBRT vs. SRS	0.59 (0.13–2.64)	0.489			0.49 (0.06–3.96)	0.503		
RT timing (reference: delayed RT) Upfront RT vs. delayed RT	**0.29 (0.08–1.03)**	**0.055**	**0.21 (0.07–0.58)**	**0.003**	**0.26 (0.05–1.36)**	**0.110**	**0.23 (0.08–0.65)**	**0.006**
Concurrent RT vs. delayed RT	**0.23 (0.08–0.61)**	**0.003**	**0.27 (0.13–0.58)**	**0.001**	**0.25 (0.08–0.82)**	**0.022**	**0.28 (0.13–0.6)**	**0.001**

Bold values means variables with significant difference (P < 0.1 for univariate cox regression and P < 0.05 for multivariate cox regression)

Concurrent delivery of ICI and RT was related to trends towards better iDPFS (HR = 2.51, 95% CI = 0.86 to 7.34, P = 0.09) **(**Fig. 1**)**. iDPFS was lower among lesions treated with not-delayed RT group (HR = 3.95, 95% CI = 1.29 to 12.14, P = 0.016) **(**Fig. 1**)**. As is illustrated in Table 2, RT timing, ds-GPA score, ECOG score, and number of brain metastasis lesions were all important factors for iDPFS on UVA, but after MVA, delayed RT was the only significant factor brought with shortened iDPFS.

### Delayed RT and response rate after radiotherapy are predictors of overall survival

At the time of the last follow up, 50.7% (37/73) of patients were alive with a median OS of 24.0 mo (range: 19.18–28.86 mo). Six-month OS rate for concurrent RT, delayed RT and upfront RT are 90.9%, 41.2%, and 100%. Twelve-month OS for these three groups are 75.4%, 29.4%, and 43.6%. Nonconcurrent iRT (HR = 2.09 95% CI = 1.08 to 4.04, P = 0.003) or delayed RT (HR = 3.67, 95% CI = 1.81 to 7.44, P < 0.001) was associated with worse OS **(**Fig. 1**)**. As displayed in Table 3, delayed RT was confirmed as an unfavorable prognostic factor for OS at MVA (HR = 3.85, 95% CI = 1.79–8.33, P = 0.001).

**Table 3:** Cox regression analysis was used to determine predictive factors for OS

Characteristics for cox regression analysis	Univariate cox regression		Multivariate cox regression	
	HR (95% CI)	p	HR (95% CI)	p
Gender (reference: female)	1.6(0.73–3.52)	0.239		
Age (reference: ≤ 65) > 65 vs. ≤ 65 years	**1.91 (0.98–3.73)**	**0.057**	1.44 (0.67–3.09)	0.347
Type of ICI treatment (reference: PD-L1)				
PD-1 vs. PD-L1	0.19 (0.03–1.47)	0.111		
Prior brain surgery (reference: no)				
Yes vs. no	0.44 (0.16–1.24)	0.122		
Number of BM (reference: ≤2 BM)				
> 2 vs. ≤2	**2.24 (1.16–4.32)**	**0.016**	**3.43 (1.17–10.07)**	**0.025**
Metastasis excluding brain (reference: no)	1.38 (0.66–2.9)	0.397		
ECOG score (reference: ECOG = 0)				
2 vs. 0	**2.68 (1.16–6.19)**	**0.021**	**5.62 (1.39–22.69)**	**0.015**
1 vs. 0	**1.97 (0.89–4.37)**	**0.094**	1.22 (0.45–3.3)	0.696
Neurological symptoms (reference: no)				
Yes vs. no	0.91 (0.47–1.75)	0.768		
Ds-GPA (reference: 0–1)				
1.5–2 vs. 0–1	0.65 (0.32–1.31)	0.226	1.68 (0.58–4.9)	0.389
2.5–4 vs. 0–1	**0.21 (0.07–0.64)**	**0.006**	1.25 (0.2–7.71)	0.812
Type of radiation				
WBRT+SRS vs. SRS	1.03 (0.47–2.27)	0.941	**0.26 (0.08–0.86)**	**0.027**
WBRT vs. SRS	**2.54 (1.14–5.69)**	**0.023**	2.37 (0.86–6.5)	0.094
RT timing (reference: delayed RT)				
Upfront RT vs. delayed RT	**0.27 (0.1–0.73)**	**0.010**	**0.14 (0.05–0.45)**	**0.001**
Concurrent RT vs. delayed RT	**0.27 (0.13–0.58)**	**0.001**	**0.24 (0.1–0.56)**	**0.001**

Bold values means variables with significant difference (P < 0.1 for univariate cox regression and P < 0.05 for multivariate cox regression)

**Fig. 2:**
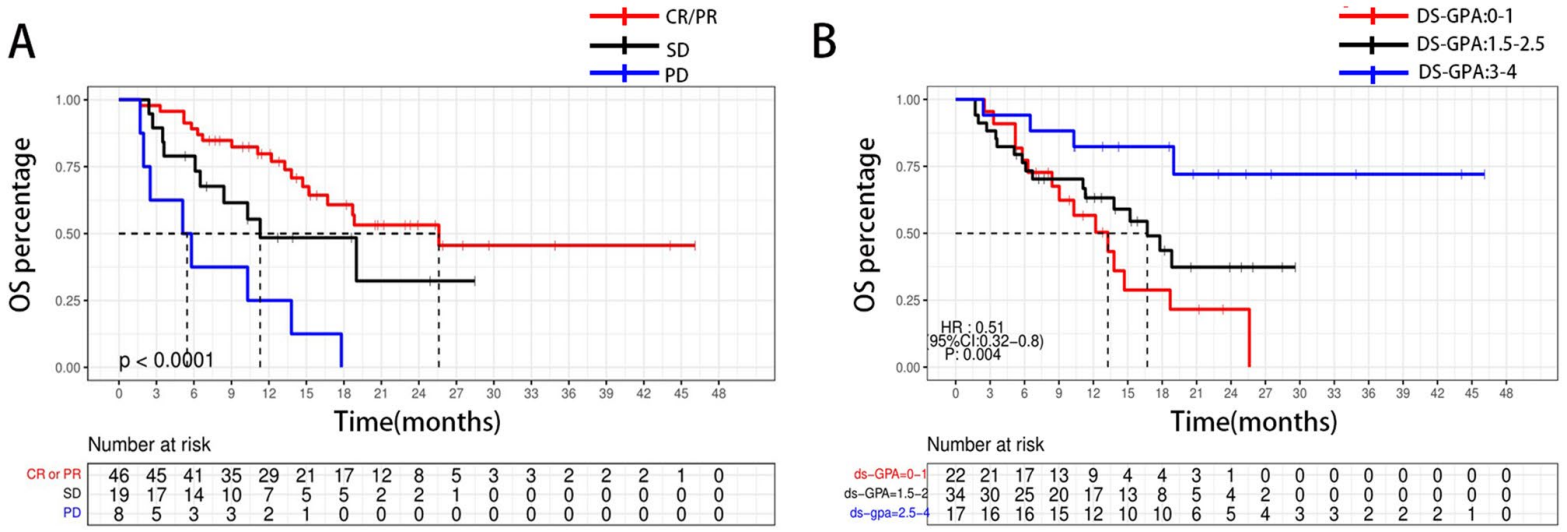
**A** OS after first brain metastasis among patients who achieved an intracranial PD, SD, or either CR or PR after RT, **B** OS after first brain metastasis among patients with ds-GPA score of 0–1, 1.5–2.5 or 3–4

Given the high intracranial responses of iRT, we further investigated the role of the first follow-up response as a prognostic factor for OS. Patients who achieved an intracranial CR or PR had significantly extended median OS (median OS, 28.37 mo for patients reached [CR/PR] vs. 15.55 mo [SD] vs. 7.37 mo [PD], P < 0.001; Fig. 2). Ds-GPA score was also related to OS (HR = 0.51, 95% CI = 0.32 to 0.8, P = 0.004), median OS was 13.4, 17.3, and 36.3 months for patients with ds-GPA scores of 0–1, 1.5–2.5, and 3–4, respectively **(**Fig. 2**).**

### Meta-analysis confirmed that delayed RT is associated with higher risk of distant brain failure rate and shorter OS

**Fig. 3:**
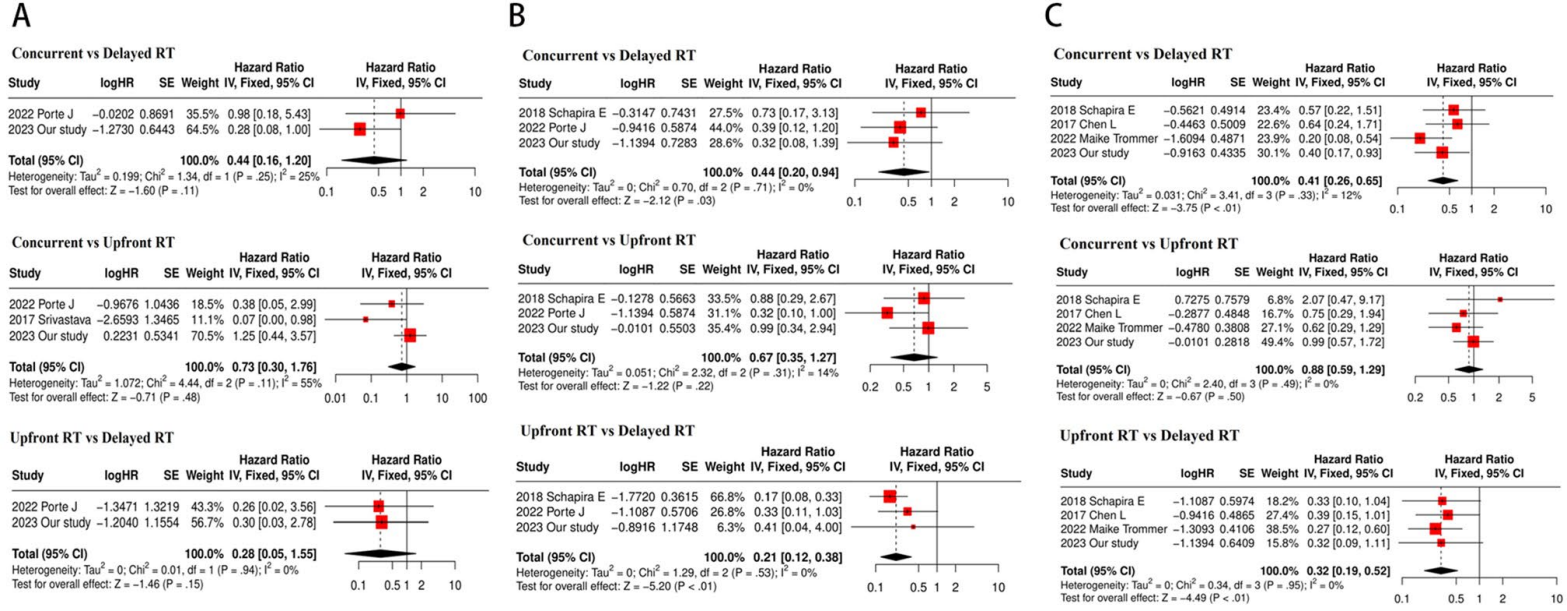
Forest plot of HR for association of sequence of RT and ICI and iLPFS (**A**), iDPFS (**B**), OS (**C**) recurrence by meta-analysis

Meta-analysis was used to determine the role of sequence in survival outcome (Fig. 3). As shown in Supplementary Tables 1, most studies use “1 month” as boundary. To minimize the selection basis, our study set with the corresponding criterion, and uses 1 month as a boundary for delayed and not-delayed RT. There were four eligible articles included, the characteristics of selected articles are summarized in Supplementary Table 2. Regarding iDPFS, combined with our study, there were totally of 37 patients in the delayed RT group, 105 patients in the concurrent group, and 52 patients in the upfront RT group. The hazard ratio for concurrent vs. delayed RT, concurrent vs. upfront RT, and upfront RT vs. delayed RT group to predict iDPFS was 0.44 (95% CI: 0.20–0.94, P = 0.03), 0.67 (95% CI: 0.35–1.27, P = 0.22), 0.21 (95% CI: 0.12–0.38, P < 0.01), respectively. There was no heterogeneity among the above comparisons and a fixed-effects model was adopted.

Combined with our study, a total of 203 patients across 3 studies reported sequence impact on OS outcome. A statistically significant survival benefit favoring the not-delayed RT group was observed (concurrent vs. delayed RT group, HR = 0.41,95% CI = 0.26 to 0.65, P < 0.01; upfront RT vs. delayed RT group, HR = 0.32,95% CI = 0.19 to 0.52, P < 0.01 respectively). As Fig. 3 shows, the treatment sequence seems unrelated to local control, but as there were only two studies enrolled, whether this is a selection bias deserves discussion.

### Shorter interval of radiotherapy and immunotherapy is associated with better best response rate of the radiated lesions

**Fig. 4:**
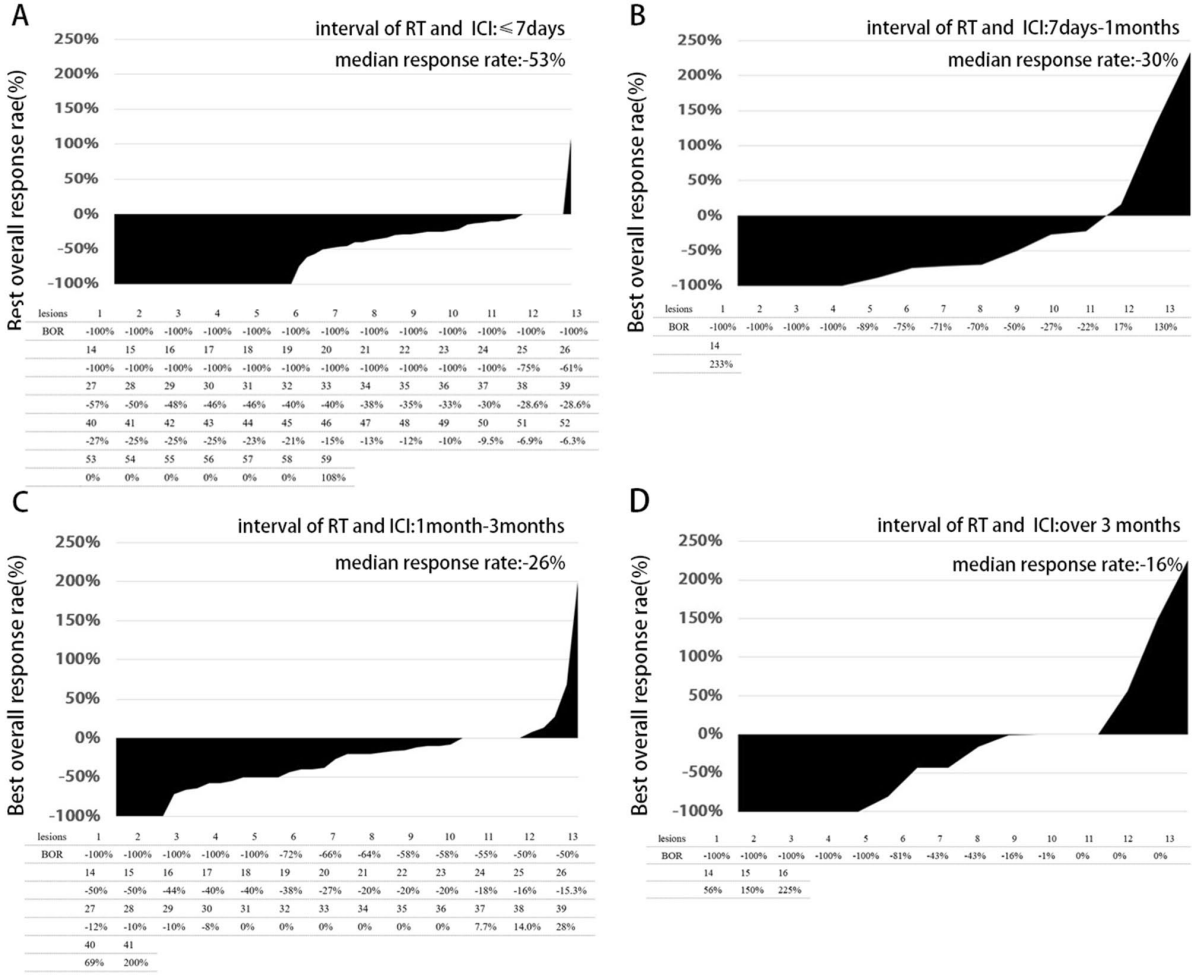
Shorter interval of RT and ICI is associated with longer local control time and better BOR. Best overall response rate of irradiated lesions in all patients when interval of RT and ICI less than 7 days (**A**), between 7 days to 2 months (**B**), over 2 months (**C**). iLPFS stratified by interval between prior ICI and RT in NSCLC patients (**D**)

A total number of 129 brain lesions visible to evaluate change on MRI were calculated with the best overall response rate (BOR). Figure 4 shows us that in those patients who receive ICIs within 7 days prior to or after RT, 40.7% (24 of 59 lesions) achieved a BOR of − 100%. For patients who receive ICI 7 days-1 month, 1–3 months, and over 3 months prior to RT, 28.6% (4 of 14 lesions), 12.2% (5 of 41 lesions), 31.3% (5 of 16 lesions) obtain a − 100% BOR. Median BOR change of the above four groups is − 53%, − 30%, − 26%, and − 16% respectively (P = 0.08). In sum, the shorter interval of RT and ICI is associated with a better best response rate of the radiated lesions.

## Discussion

In this study, we evaluate the outcome of 73 NSCLC patients who receive ICIs and RT to BMs, and investigate the best sequence. While retrospective series have explored this topic in melanoma patients receiving ipilimumab and RT [16], few have reported patient outcomes in NSCLC patients with anti-PD-1 ICI in BMs, and our series represents one of the most comprehensive analyses to date. This study provides several important findings. First, in terms of sequencing of therapy, not-delayed RT maximizes the benefits of synergistic therapy. Second, the benefit of combined modality therapy is maximized when immediate intracranial RT is delivered to ICI naive lesions. Third, not only is the high intracranial response rate in patients undergoing RT and immediate ICI impressive, but early evaluation of this response is predictive of OS. It is important to note that given the difficulty in conducting prospective clinical trials, these results are crucial to optimal patient management.

Numerous studies have demonstrated the survival advantages of RT and ICIs together [1]. Studies on patients with BMs found that RT might change blood–brain barrier functioning and permit the entry of immune cells and ICI into the brain [17–19]. Recent studies recommend RT combined with ICI to treat patients with BMs [20, 21]. Institutional studies in melanoma with BMs have reported the impact of iRT sequence on survival outcome [22, 23], but few have specifically investigated impact in lung cancer. In our study, which included 73 NSCLC patients with BMs, we discovered that the delayed RT group had inferior survival outcomes, including iLPFS, iDPFS, and OS. Similarly, Schapira and colleagues reported declined iDPFS and OS in 37 patients with delayed RT (defined as within 1mo or 1mo prior to ICIs) [24]. Our study supports their findings in a larger sample size, and provides multiple with more observations, including lesions-specific objective response rate. A large international meta-analysis was published in 2019 [25], this study enrolled melanoma and lung cancer patients with BMs, and revealed that delayed RT predicts the worst OS (1-year OS was 40.7%, 56%,65% for delayed RT, concurrent RT, and upfront RT, P = 0.00045), but there are only 19 BM lesions originated from lung cancer. In keeping with their finding that RT shouldn’t be delayed, our meta-analysis increased the sample size and solely recruited lung cancer patients with BM.

Many trials, including those done by Schapira E et al., Porte J et al., Maike Trommer et al., and Hubbeling et al. used “1 month” as the threshold for “concurrent RT” [24, 26–28]. In these trials, patients administered with concurrent iRT experienced better survival outcomes than those treated with non-concurrent therapy. The definition of “concurrent ICI” ranges from “within 1 week” [29] to “5.5 months” [30]. Some studies suggest ICI’s half time prior to or after RT as a definition for “concurrent therapy”, which is based on small samples on retrospective studies [13, 7, 31]. We use “x-tile” software, there is a sustaining benefit in iLPFS and OS of “not delayed RT” when we compress the time interval between upfront ICI and following RT from “1 month” to “14 days”. This is the reason why use two weeks as the boundary between concurrent and non-concurrent RT. Similarly, Chen found that patients who received ICI more than two weeks prior to stereotactic radiation had lower OS than the contemporaneous group [32]. Imber et al. also use “2 weeks” as a boundary for delayed and not-delayed RT [33]. Numerous studies have identified and assessed biomarker alterations, such as those in mannose-6-phosphate (MPR) [34], tumor-reactive T cells [35], and PD-L1 [35], to assist in determining the ideal time to provide anti-PD-1/L1 therapy. Hettich’s work shows that ICI is effective early after RT-mediated T-cell induction since CD8+T-cell count peaks after 5 days post-RT and subsequently drops to pre-RT levels following 6Gy 2 fractions [36]. Frey’s work reinforces this finding, following tumor irradiation with 10Gy/2F, it was shown that CD8+T-cells peak at day 8 and decline significantly by day 9 [37]. Together, these findings imply that the optimal definition of “concurrent RT” might be reached by closely monitoring tumor-reactive T cells after radiotherapy.

Patients who received ICIs exhibited prolonged local control and had superior BOR when the gap between iRT and RT was shorter (P = 0.0544). We recommend shortening the time between RT and ICI; in other words, ICI administration schedules shouldn’t be changed while RT is being administered. Other authors found a similar result in favor of immediate delivery. Italian Association of Radiotherapy and Clinical Oncology (AIRO) conducted a sizable multi-institutional retrospective analysis, they showed significant differences when comparing OS in patients treated with interval of iRT ≤ 7 days to those with interval > 7 days (P = 0.007), in favor of short interval group [38]. In contrast to non-immediate ICI, Kotecha found that patients who received immediate ICI had superior BOR [13]. Innovatively, Dagoglu’s case series evaluation revealed that 95.8% of abscopal responses happened in patients who underwent RT right away after immunotherapy [39]. For preclinical studies, Dovediin’s study revealed that anti-PD-L1 drugs started seven days after RT were related to worse survival outcomes than those started on the first or last day [35]. Some studies demonstrated that very close intervals of RT and anti-PD-1 will be able to potentiate early T-cell activation stimulated by RT [21, 40], to some extent accounting for our results.

The relevant limitation is its retrospective nature with a high risk of selection bias, however, we have tried to exclude the strong confounding factors. We only enrolled BM patients from NSCLC and excluded those who have received previous brain radiotherapy. Furthermore, only those who received PD-1/PD-L1 were included. Drawbacks of this study were: firstly, follow-up time bias, as patients who underwent delayed RT were likely to have a much closer frequency of screening MRI and close follow-up. Secondly, even though the baseline characteristics were comparable across groups, patients were not randomly chosen to undergo ICI, stereotactic radiotherapy, or WBRT as their initial course of treatment. Thirdly, chromosomal abnormalities and PD-L1/PD-1 expression were not examined since a significant fraction of patients’ conditions were unknown. Fourth, delayed RT is frequently used for BM progression. These patients are likely to have ICI resistance, and this imbalance might affect the outcome. Fifth, despite our efforts to include recruited patients from two centers and conduct a meta-analysis to improve patient volume, only seventeen patients (23.3%) underwent delayed RT in our study, the small number of patients negatively impacted the outcome of this study.

## Conclusion

Delayed RT is associated with worse survival outcome including iLPFS, iDPFS and OS in NSCLC, we suggest “2 weeks” as the boundary between delayed and not-delayed RT. Shorter interval between ICI and RT is associated with longer local control time and better brain response rate.

### Supplementary Information

Below is the link to the electronic supplementary material.

Supplementary Figure 1: Flow chart of patient inclusion (JPG 115.5 kb)

Supplementary Figure 2: PRISMA flow diagram (JPG 118.0 kb)

Supplementary Figure 3: Impact of different interval of iRT on iLPFS in NSCLC patients (JPG 682.0 kb)

Supplementary Figure 4: Impact of different interval of iRT on iDPFS in NSCLC patients (JPG 603.7 kb)

Supplementary Figure 5: Impact of different interval of iRT on OS in NSCLC patients (JPG 648.5 kb)

Supplementary material 6 (DOCX 41.1 kb)

Supplementary material 7 (DOCX 17.5 kb)

## Data Availability

The raw data supporting the conclusions of this article will be made available by the authors, without undue reservation.
