# Supplementary figures and images for: Improved survival outcome with not-delayed radiotherapy and immediate PD-1/PD-L1 inhibitor for non-small-cell lung cancer patients with brain metastases
Source: J Neurooncol. 2023 Oct 17;165(1):127–37. doi: 10.1007/s11060-023-04459-4 (PMC10638122; doi:10.1007/s11060-023-04459-4)

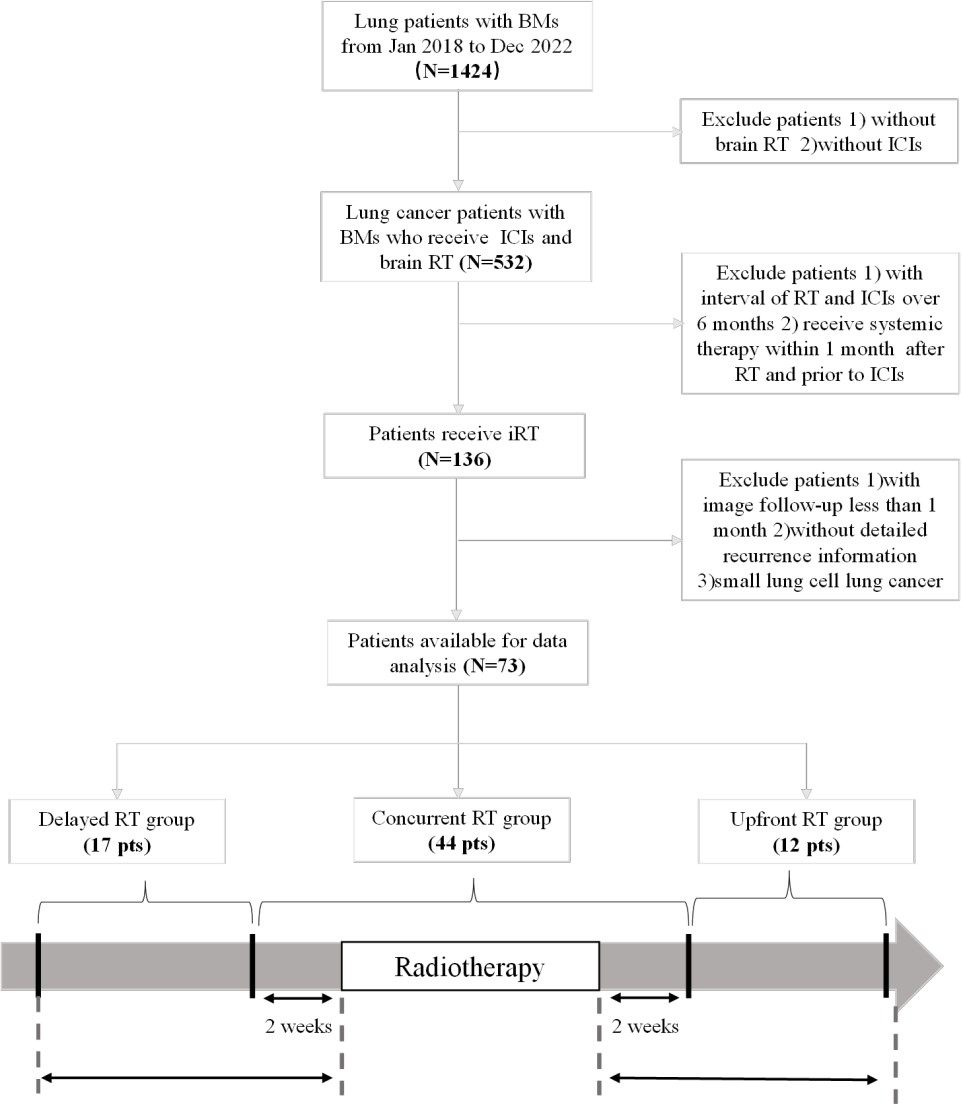

Supplement: Supplementary file 1 — Supplementary Figure 1: Flow chart of patient inclusion (JPG 115.5 kb) [file 11060_2023_4459_MOESM1_ESM.jpg]

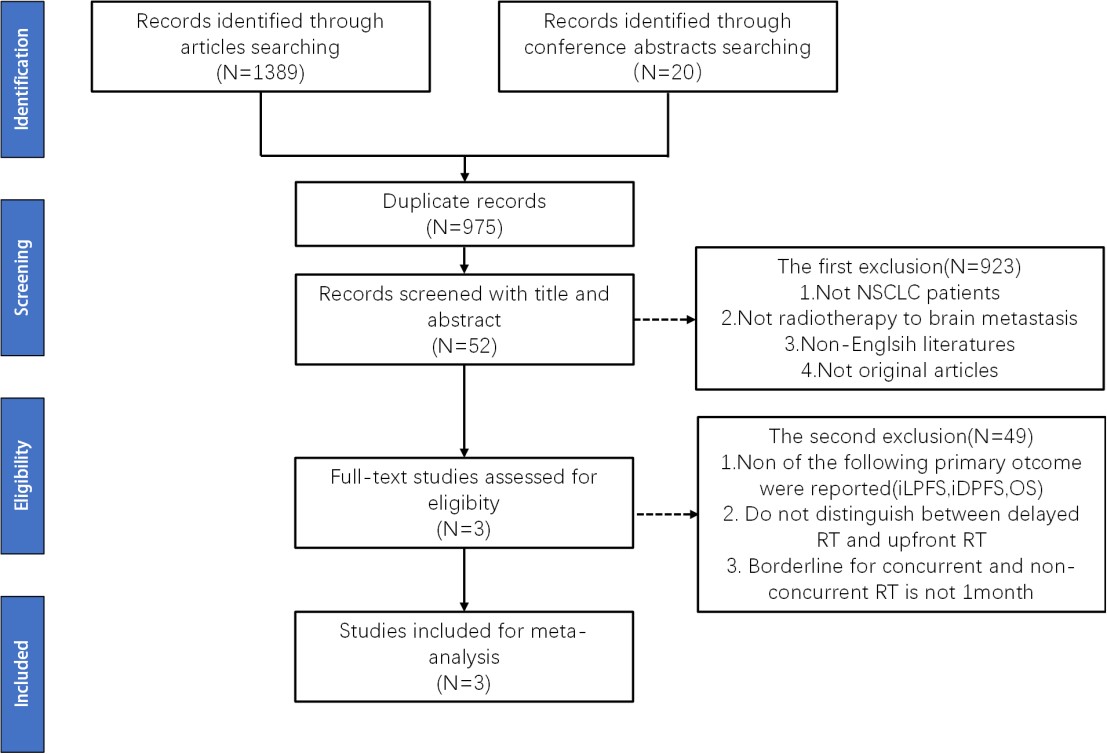

Supplement: Supplementary file 2 — Supplementary Figure 2: PRISMA flow diagram (JPG 118.0 kb) [file 11060_2023_4459_MOESM2_ESM.jpg]

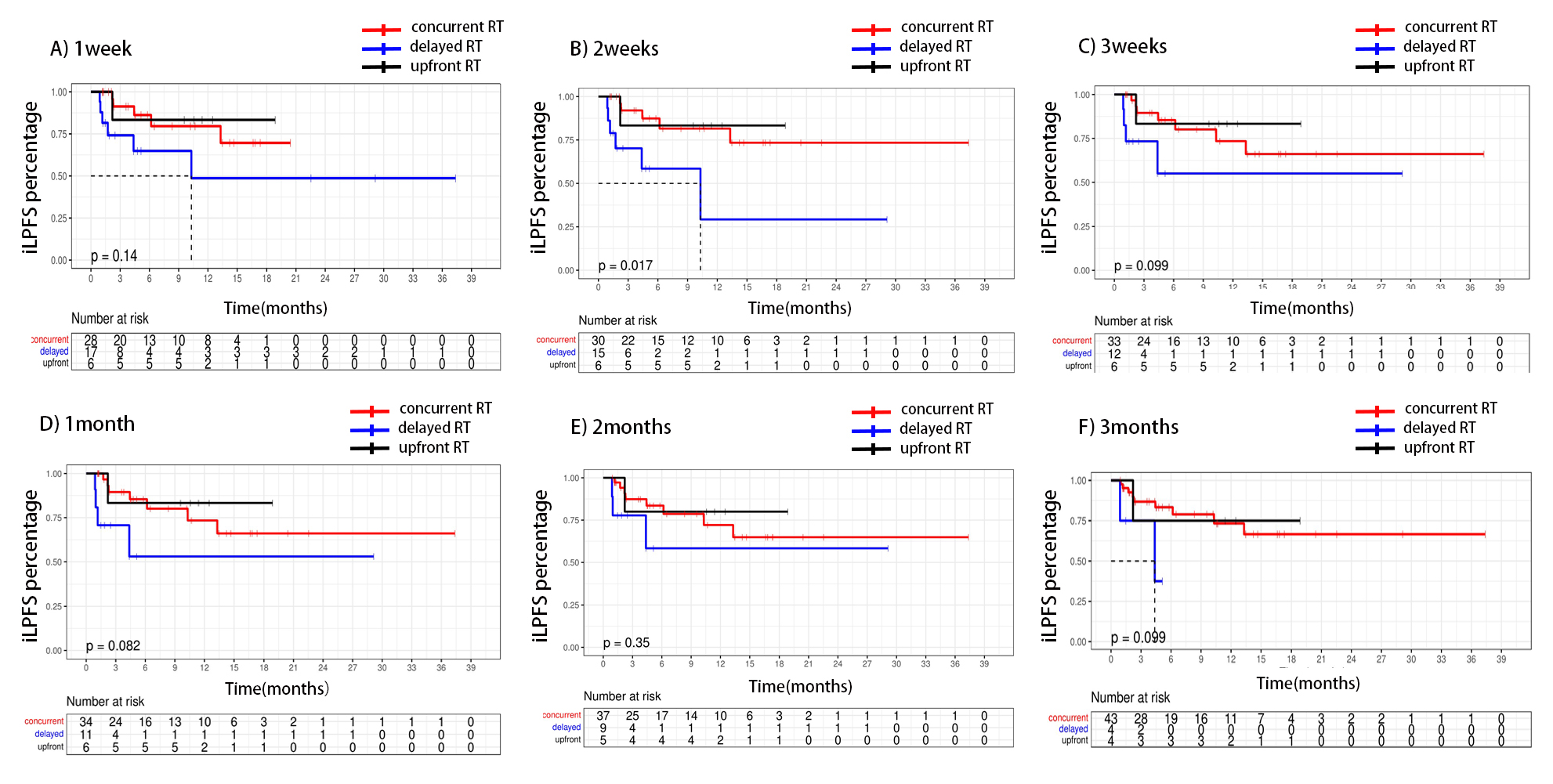

Supplement: Supplementary file 3 — Supplementary Figure 3: Impact of different interval of iRT on iLPFS in NSCLC patients (JPG 682.0 kb) [file 11060_2023_4459_MOESM3_ESM.jpg]

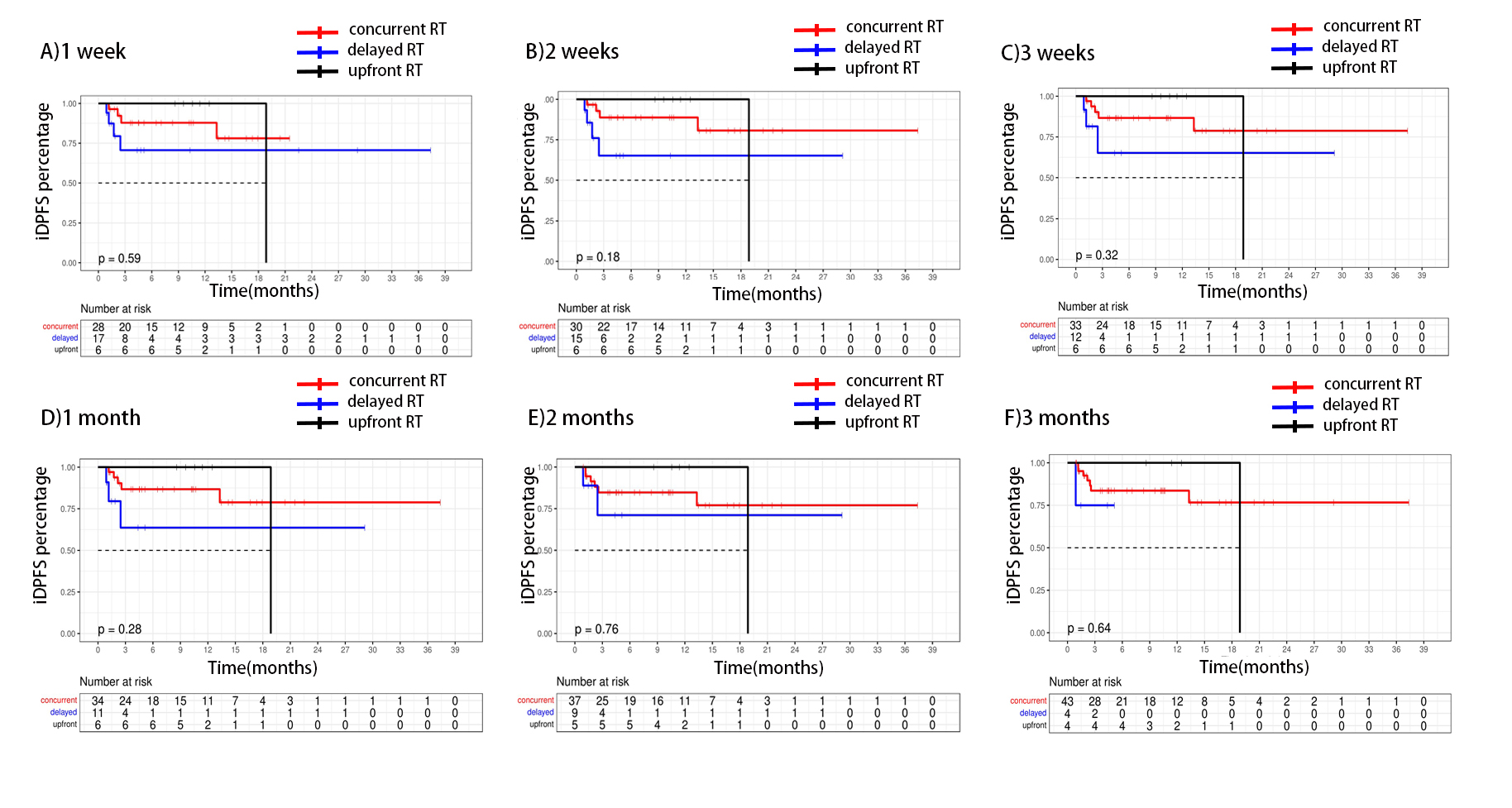

Supplement: Supplementary file 4 — Supplementary Figure 4: Impact of different interval of iRT on iDPFS in NSCLC patients (JPG 603.7 kb) [file 11060_2023_4459_MOESM4_ESM.jpg]

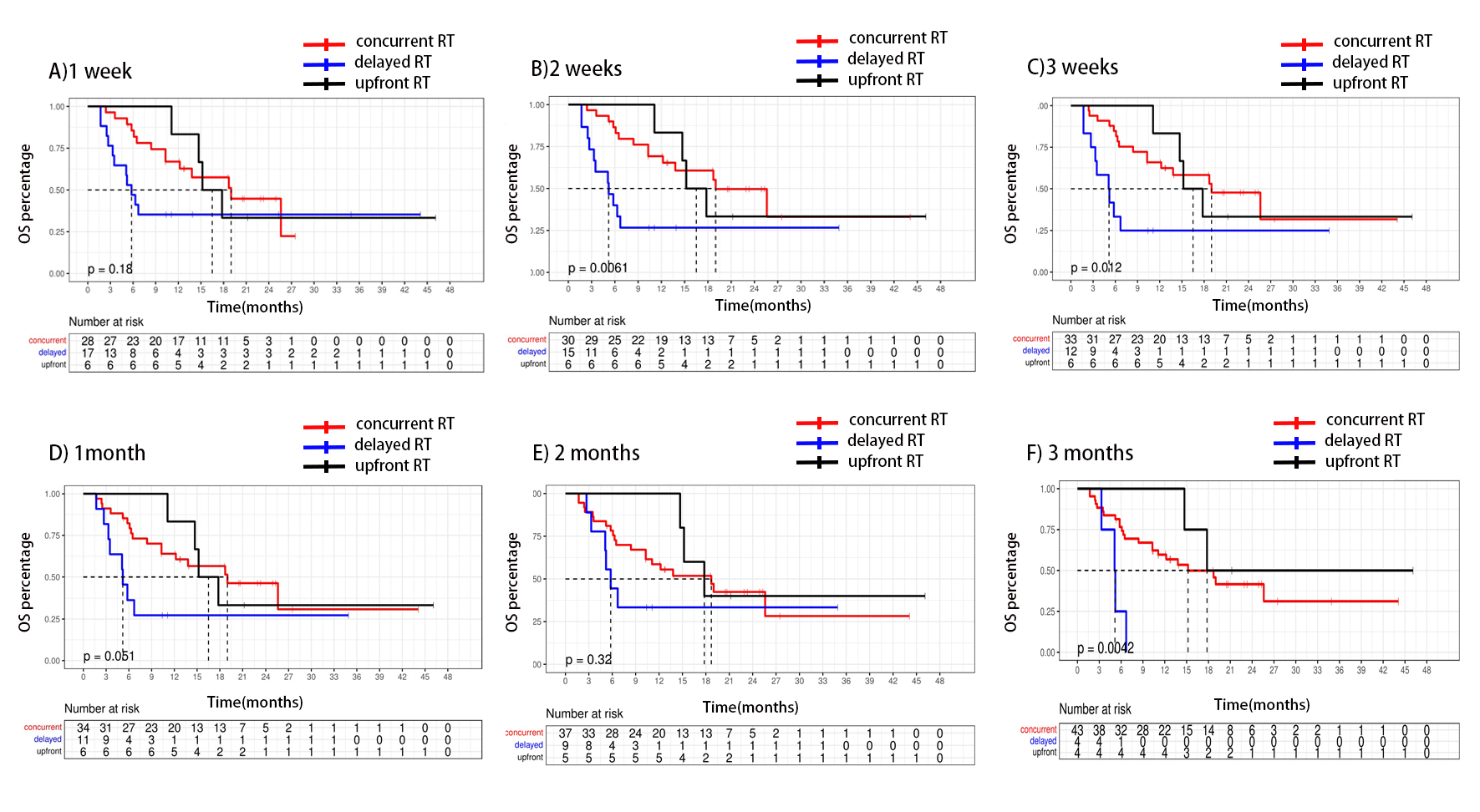

Supplement: Supplementary file 5 — Supplementary Figure 5: Impact of different interval of iRT on OS in NSCLC patients (JPG 648.5 kb) [file 11060_2023_4459_MOESM5_ESM.jpg]
