# Supplementary material for: Improved survival outcome with not-delayed radiotherapy and immediate PD-1/PD-L1 inhibitor for non-small-cell lung cancer patients with brain metastases
Source: J Neurooncol. 2023 Oct 17;165(1):127–37. doi: 10.1007/s11060-023-04459-4 (PMC10638122; doi:10.1007/s11060-023-04459-4)
Supplement: Supplementary file 6 — Supplementary material 6 (DOCX 41.1 kb) [file 11060_2023_4459_MOESM6_ESM.docx]

**Supplementary table1: Review and primary outcome of studies focused on impact of iRT sequence on survival outcome.**

| **Author** | **Patients enrolled** | **definition** | **Baseline characters** | **Main outcome** | **Main conclusion** |
| --- | --- | --- | --- | --- | --- |
| Schapira E 2018[25] | 37 NSCLC | Cutoff: 1 month | Most lesions were treated with  18Gy in a single fraction (n=61; 71.8%). | **RT→ICI (n=24) vs RT=ICI (n=8) vs ICI→RT (n=5)**   - 1-y OS: 70% vs 87.3% vs 0% (P=0.008) - 1-y R-PFS: 34.2% vs 61.5% vs 0% (P=0.042)   **ICI→RT + RT=ICI vs RT→ICI**   - 1-y L-PFS: 100% vs 72.3% (P=0.016) - No patient experienced grade≥4 toxicity | - Patients treated with RT=ICI was associated with favorable OS and loco-regional disease control - Patients treated with ICI**→**RT might evolve a more aggressive disease with limited survival, regardless of further local intracranial intervention |
| Porte J 2022[28] | 51 patients with 84 BM from NSCLC | Cutoff: 1 month | Most lesions were treated with 15-21Gy /1F (56.0%)  or 18-27Gy/3F (41.8%) | **RT→ICI (n=18) vs RT=ICI (n=46) vs ICI→RT (n=20)**   - 1-y R-PFS:22.8 % vs 48% vs 24.1% (P = 0.031) (after excluding Durvalumab group) - 1-y L-PFS: 78.9% vs 70.1% vs 77.8% (P = 0.79) - RT=ICI did not induce more acute neurologic toxicity (P = 0.66) | - RT=ICI group offer the best locoregional control, without increasing the risk of toxicity, compared to RT→ICI or ICI→RT |
| Kotecha 2019[15] | 150 patients (including 99 lung cancer) underwent SRS to 1003 BM | Immediate（Concurrent） ICI:±within 1(5) biological half-lives of  the date of SRS |  | **ICI pre-exposed lesions (n=196) vs ICI naïve lesions (n=553)**   - ICI pre-exposed lesions had lower response rate compared with ICI naive lesions (45% vs 63% lesion reduction, p＜0.001) - The best response rates were observed in ICI naive lesions that underwent treatment with SRS and immediate ICI (−100%, P < 0.001). - The timing of therapy did not adversely affect the risk of treatment-related toxicity | - Sequencing of ICI around SRS is associated with overall response, best response, and response durability, - Most substantial effect is in ICI naive BM undergoing immediate combined modality therapy |
| Chen L 2017[34] | 260 patients (including 157 NSCLC) with 623 BMs | Cutoff: 2 weeks | median SRS-SRT dose was 20Gy in 1 fraction | **RT→ICI (n=30) vs RT=ICI (n=28) vs ICI→RT (n=23)**   - 1-y OS: 15.0 mo vs 24.7 mo vs 12.0 mo - OS for RT→ICI (P=0.002; HR,3.82) and ICI→RT (P=0.021; HR, 2.64) was associated with worse OS compared with RT=ICI - Concurrent ICI predicted reduced probability of subsequent development of ≥3 new BMs (P=0.045; OR, 0.337) | - RT=ICI is associated with a reduced incidence of new intracranial metastases and favorable survival outcomes |
| Srivastava 2017[43] | 50 patients (24 NSCLC) | Cutoff: 3 weeks | na | **RT=ICI (n=27) vs RT→ICI (n=23)**   - 6-month LPFS: 100% vs 76% (P=0.04) - 6-month RPFS: 71% vs 41%(P=0.02)   **In NSCLC, RT=ICI vs RT→ICI**   - Improved RPFS in RT=ICI group (P=0.04) | - RT=ICI appears to improve LC and DBC when compared to ICI→RT |
| Scoccianti 2021[40] | 100 NSCLC treated with ICI | Cutoff: 7 days | ＞18Gy/1F(48%),  ≤18Gy/1F(12%),  Any Gy/more fractions (40%) | **Interval between RT-ICI ＞7days(n=10) vs ≤7days(n=90)**   - OS: shorter OS for ＞7 days group (propensity score-adjusted HR 6.97, CI 1.64–29.52; P =0.008) - Any grade radionecrosis:21.1% vs 10% (P=0.68)   **received IT at the diagnosis of BM: RT→ICI (n=31) vs ICI→RT(n=28)**   - RT**→**ICI was predictive for better OS compared to ICI→RT, but the difference was not significant (propensity score-adjusted HR=0.74, CI 0.25–2.15; P =0.574) | - IT schedule should not be modified when patients receive SRT - advantage for administering SRT before giving IT |
| Imber 2017[35] | 45 NSCLC | Cutoff: 2 months | 91% SRS (median dose=21Gy), 9% hRT (median dose=30Gy/5F) | **RT=ICI (n=29) vs ICI**→**RT (n=16)**   - median time to distant brain failure (DBF): 4.9 vs. 3.9 months, P=0.6) - lesional response: concurrent (CR 9%, PR 50%, S 32%, PD 9%) vs. sequential (CR 10%, PR 31%, S 48%, PD 10%) (no difference) | - Did not identify an advantage to concurrent vs. sequential RT |
| Li 2020[30] | 13 NSCLC (prospective) | Cutoff: 7 days | SRS (dose unknown) | - Median intracranial PFS time was 9.7 months - 4-mo intracranial PFS rate was 75% - Extracranial objective response rate was 33% in the 12 evaluable pts - median response duration of 9.1 months | - Concurrent SRS with ICI was safe for pts with active NSCLC brain metastasis - Preliminary analyses of efficacy were encouraging for durable intracranial and extracranial response |
| Maike Trommer 2022[27] | 93 patients with 319 BMs (22.8% lung) | Cutoff: 1month | Mean BED was 55.7 ± 10.1Gy | **RT→ICI (n=19) vs RT=ICI (n=63) vs ICI→RT (n=11)**   - 1-y OS:47.1% vs 58.1% vs 18.2% (P＜0.001) - 2-y OS:47.1% vs 44.5% vs 9.1% (P＜0.001) - 3-y OS:41.2% vs 34.2% vs 9.1% (P＜0.001) | - Timing appears to be an important factor for OS, with the best results obtained when RT was delivered before or during IC |
| Hubbeling 2018[29] | 50 NSCLC | Cutoff: 1 month | 70% receive SRS, 16% receive PBI, 58% receive WBRT | **RT→ICI (n=21) vs RT=ICI (n=14) vs ICI→RT (n=5) (lesions, SRS)**   - Grade 3-4 AEs: 10% vs 7% vs 0%(P=0.27) - Symptomatic TRIC: 29% vs 36% vs 20%(P=0.90)   **RT→ICI (n=18) vs RT=ICI (n=6) vs ICI→RT (n=5) (lesions, WBRT)**   - Grade 3-4 AEs: 6% vs 33% vs 0%(P=0.17) - Symptomatic TRIC: 17% vs 33% vs 0%(P=0.51) | - There were no differences in rates of any grade AEs or of grade ≥ 3 AEs based on the sequencing of RT/ICI. - Rates of symptomatic treatment-related imaging change (TRIC) were similar irrespective of RT/ICI timing |
| Cabanie 2021[44] | 59 patients with 103 brain metastases (NSCLC 60%) | A:≤7 days  B:8-14 days  C:15-30 days | GTV: 20Gy/1F or 33Gy/3F  PTV: 14Gy/1F, or 23.1Gy/3F | **Group A (n=11) vs B (n=31) vs C (n=49)**   - 1-y LPFS: 76% vs 76% vs 83% (P＞0.4) - time lapse between concomitant immunotherapy and HFSRT was not a significant predictive factor of LC (P = 0.133 in ICI→RT and P = 0.859 in RT→ICI) | - time-lapse between immunotherapy and SRT was not a significant predictor of LC |
| Ahmed 2017[45] | 17 patients with 49 brain metastases | Within 6 months | 18-24Gy/1F (82% lesions), 25Gy/5F (18% lesions) | **ICI→RT (n=13 lesions) vs RT→ICI (n=22 lesions) or RT=ICI (n=14 lesions)**   - 6-month RPFS: 0% vs 57% (P=0.05) - OS univariate analysis: HR=9.2(95% CI=1.9-65.3, P=0.006) - OS multivariate analysis: HR=3.6(95% CI=0.74-26.9, P=0.11) | - Prospective evaluation to determine how these two modalities can be used to improve distant brain control and OS is warranted. |
| Our study 2023 | 73 NSCLC | Cutoff: 2 weeks | RT | **ICI→RT (n=17) vs RT=ICI (n=44) or RT→ICI (n=12)**  Delayed RT brought worse iLPFS (P=0.0029), iDPFS (P=0.0163) and OS(P＜0.001) compared to not-delayed RT group (RT=ICI or RT→ICI group) | - We suggest patients with ICI naive BM undergoing combined immunotherapy within 2 weeks. - ICIs should be delivered as close to SRS as possible. |

**Supplementary table2: Characteristics of studies included in the meta-analysis**

| **Study** | **Enrolled patients** | **Country** | **Score** | **Sample Size** | **HR (95% CI) for comparison of three groups** | | | |
| --- | --- | --- | --- | --- | --- | --- | --- | --- |
|  |  |  |  |  | **Outcome** | **concurrent RT vs delayed RT** | **concurrent RT vs upfront RT** | **upfront RT vs delayed RT** |
| Schapira E 2018[25] | 37 NSCLC patients | American | 10 | Delayed RT:5  Concurrent RT:8  Upfront RT: 24 | iLPFS | UN | UN | UN |
|  |  |  |  |  | iDPFS | 0.73(0.17-3.13) | 0.88(0.29-2.67) | 0.17(0.08-0.33) |
|  |  |  |  |  | OS | 0.57(0.2—1.51) | 2.07(0.47-9.17) | 0.33(0.1-1.04) |
| Porte J 2022[28] | 51 NSCLC patients | France | 9 | Delayed RT:20  Concurrent RT:46  Upfront RT:18 | iLPFS | 0.98(0.18-5.43) | 0.38(0.05-2.99) | 0.26(0.02-3.56) |
|  |  |  |  |  | iDPFS | 0.39(0.12-1.20) | 0.32(0.1-1.0) | 0.33(0.11-1.03) |
|  |  |  |  |  | OS | UN | UN | UN |
| Maike Trommer 2022[27] | 93 patients with BMs (21 NSCLC) | Germany | 8 | Delayed RT:11  Concurrent RT:63  Upfront RT:19 | iLPFS | UN | UN | UN |
|  |  |  |  |  | iDPFS | UN | UN | UN |
|  |  |  |  |  | OS | 0.2(0.08-0.54) | 0.62(0.29-1.29) | 0.27(0.12-0.6) |
| Our study 2023 | 73 NSCLC patients | Chi1na | 9 | Delayed RT:12  Concurrent RT:51  Upfront RT:10 | iLPFS | 0.28(0.08-1.00) | 1.25(0.44-3.57) | 0.30(0.03-2.78) |
|  |  |  |  |  | iDPFS | 0.32(0.08-1.39) | 0.99(0.34-2.94) | 0.41 (0.04-4.00) |
|  |  |  |  |  | OS | 0.40 (0.17-0.93) | 0.99(0.57-1.72) | 0.32 (0.09-1.11) |

UN=unknown, **1month as cutoff**
