# Supplementary material for: Improved survival outcome with not-delayed radiotherapy and immediate PD-1/PD-L1 inhibitor for non-small-cell lung cancer patients with brain metastases
Source: J Neurooncol. 2023 Oct 17;165(1):127–37. doi: 10.1007/s11060-023-04459-4 (PMC10638122; doi:10.1007/s11060-023-04459-4)
Supplement: Supplementary file 7 — Supplementary material 7 (DOCX 17.5 kb) [file 11060_2023_4459_MOESM7_ESM.docx]

**Supplementary text1: Publication Search and Inclusion for Meta-Analysis**

We searched PubMed, Embase and ClinicalTrials. gov databases for articles. The last search update was September 2023, using the search terms (“lung cancer” or “lung carcinoma” or “lung tumor” or “NSCLC” or “non-small cell lung cancer”) AND (“radiotherapy” OR “RT” OR “radiosurgery” ) AND (“brain metastases” OR “brain metastasis”) AND (“immunotherapy” OR “checkpoint inhibitor”). Additionally, we searched conference abstracts from the European Society for Medical Oncology, the American Society of Clinical Oncology. Articles with the following features were included: i. include non-small-lung cancer patients with brain metastasis; ii. treated with brain RT and ICI, either concurrent or non-concurrent; iii. At least one of the following primary outcomes were reported: intracranial local progression free survival(iLPFS), intracranial distant progression free survival(iDPFS); and OS. Exclusion criteria were as follows: i. Case reports, reviews, or letters; ii. Studies with overlapping or repeating data; iii. Published articles that they were not written in English; iv. Do not distinguish between delayed RT and upfront RT, or blend into non-concurrent RT; v.do not use 1 month as borderline between concurrent and non-concurrent RT.

**Supplementary text2: Methodology details the statistical analysis**

Comparisons between patient characteristics were performed by using one-way analysis of variance. Cox proportional hazards models were used for univariate analysis, those with p≤0.1 were finally included in multivariate Cox proportional hazards models. We also use Kaplan-Meier analysis to study the impact of RT/ICI sequence on iLPFS, iDPFS, and OS. A two-sided p< 0.05 was considered statistically significant for the analyses. X-tile program was adopted to acquire optimal cutoff points of delayed RT.

If survival comparison information was present only in figures, two reviewers would use Engauge Digitizer 10.8 to collect data from the statistical graphs independently, mean values would be adopted [14]. A study's heterogeneity was assessed by Q-test and statistical inconsistency index (I^2^) for each outcome. Random-effects model was applied when P＜0.05 (for the Q-test) or I^2^≥50%. Otherwise, a fixed-effects model was applied.

Above analyses were performed using R software, version 4.2.2 (R Foundation for Statistical Computing).
